# Hospitals and the Insurance Bill

**Published:** 1911-09-30

**Authors:** Godfrey H. Hamilton

**Affiliations:** Secretary National Hospital for Paralysed and Epileptic, Queen Square. 16 Madrid Road, Barnes, S.W.


### HOSPITALS AND THE INSURANCE BILL.

*To the Editor of* The Hospital.

Sir,—It is generally admitted by those closely associated with our large medical charities that the National Insurance Bill will have an adverse effect upon the income of hospitals; but while the Bill may injure the hospitals in this way, might it not, by improving their status, be the means of doing something for hospital workers?

There are no available statistics, but basing the statement on the wages sheets of some hospitals known to me, I think there must be at least 6,000 employees of London hospitals, about three-quarters of whom are women, who would come within the Act. On this basis, a rough calculation shows that the amount to be raised by taxation will be over £10,000 a year.

At the present time, during sickness of moderate duration, the hospitals give their employees free treatment, either in or out of the wards. Where, then, except in cases where workers become permanently ill, is the medical benefit of the Act, and what are the London hospitals and their staffs going to get for £10,000?

But the Bill provides for the formation of approved societies, and the formation of a society or societies, in the case of hospitals, is easy. In order to qualify, it would be necessary to ask the constituent hospitals, in any combined scheme where the total of workers is under ten thousand, each to guarantee to subscribe a small annual sum—two or three guineas would no doubt meet the case.

The members of such a society would be in an excellent position. They would get medical treatment by the most skilled hands it is possible to engage, and this benefit would start as soon as required, other insured persons having to wait six months before becoming entitled to it.

As the hospitals, to help such a scheme, would no doubt continue to give treatment free to members, the larger part of the grant receivable from the Government (which, I take it, would be the £10,000 returned, minus contributions for sanatorium benefits—1s. 3d. per head per annum) would go into the coffers of the approved society.

What a chance to start the superannuation scheme so badly needed by hospitals!

At present a hospital worker who has spent the years of his service in more than one hospital can only look to his last employer for help in old age. And he, and another who has spent all his working days in one service, as we are at present placed, when they retire must rely upon the good feeling of the Committee of Management who happen to be in office. But the ideas of Committees vary greatly on the subject of pensions. In the report of one of our most important London hospitals for last year is recorded that thirty years' service of the retiring secretary was rewarded by a pension of £75 a year. In contrast to this almost incredible meanness is found, happily, fairer and more humane treatment of employees by other Committees of Managament.

In any case, the position at present for the worker nearing the time of rest is far too uncertain. Now, thanks to Mr. George's Bill, there is a chance of a scheme being brought forward which, while including all the benefits of the measure and others of equal importance to the servants of our hospitals, prevents the unproductive expenditure of over £10,000 a year.—I am, sir, your obedient servant, Godfrey H. Hamilton, Secretary National Hospital for Paralysed and Epileptic, Queen Square.

16 Madrid Road, Barnes, S.W.
September 25.

